# A Diagnostic Model Using Exosomal Genes for Colorectal Cancer

**DOI:** 10.3389/fgene.2022.863747

**Published:** 2022-07-15

**Authors:** Tianxiang Lei, Yongxin Zhang, Xiaofeng Wang, Wenwei Liu, Wei Feng, Wu Song

**Affiliations:** ^1^ Department of Gastrointestinal Surgery, The First Affiliated Hospital, Sun Yat-sen University, Guangzhou, China; ^2^ Laboratory of General Surgery, The First Affiliated Hospital, Sun Yat-sen University, Guangzhou, China; ^3^ Center for Digestive Disease, The Seventh Affiliated Hospital of Sun Yat-sen University, Shenzhen, China

**Keywords:** exosome, diagnostic model, functions, colorectal cancer, bioinformatics analysis

## Abstract

Colorectal cancer (CRC) is a leading cause of cancer-related deaths worldwide. Exosomes have great potential as liquid biopsy specimens due to their presence and stability in body fluids. However, the function and diagnostic values of exosomal genes in CRC are poorly understood. In the present study, exosomal data of CRC and healthy samples from the exoRBase 2.0 and Gene Expression Omnibus (GEO) databases were used, and 38 common exosomal genes were identified. Through the least absolute shrinkage and selection operator (Lasso) analysis, support vector machine recursive feature elimination (SVM-RFE) analysis, and logistic regression analysis, a diagnostic model of the training set was constructed based on 6 exosomal genes. The diagnostic model was internally validated in the test and exoRBase 2.0 database and externally validated in the GEO database. In addition, the co-expression analysis was used to cluster co-expression modules, and the enrichment analysis was performed on module genes. Then a protein–protein interaction and competing endogenous RNA network were constructed and 10 hub genes were identified using module genes. In conclusion, the results provided a comprehensive understanding of the functions of exosomal genes in CRC as well as a diagnostic model related to exosomal genes.

## Introduction

Colorectal cancer (CRC) is one of the most common malignant tumors of the digestive tract and presents the second-highest mortality rate (9.2%) among all cancers (Arnold et al., 2017; Bray et al., 2018). Tumor metastases and invasion are associated with poor prognosis and lead to a low five-year survival rate in CRC patients (Siegel et al., 2018). CRC is typically detected by colonoscopy, measuring carcinoembryonic antigen (CEA) levels, multitarget stool DNA testing, and the septin 9 gene methylation blood test (Ahluwalia et al., 2021). Although colonoscopy is a highly sensitive method, it is invasive and uncomfortable for patients and its accuracy depends on the skill level and experience of the endoscopist (Schreuders et al., 2015). CEA levels have been widely used as tumor markers for the detection of CRC. However, it is still limited in terms of sensitivity and specificity. Therefore, there is an urgent need to identify more effective and less invasive surrogate CRC-specific diagnostic markers for the rapid, noninvasive, and high sensitivity screening of patients.

The use of exosomes as a new noninvasive approach for diagnosing diseases has attracted growing attention (Mousavi et al., 2019). Exosomes are extracellular vesicles containing messenger RNAs (mRNA), microRNAs (miRNA), long noncoding RNAs (lncRNA), circular RNAs (circRNA), DNA, lipids, and proteins with sizes between 40 and 150 nm and density between 1.13 and 1.19 g/ml (Yáñez-Mó et al., 2015; Shi et al., 2021). Notably, exosomal biomarkers and molecule information remain relatively stable in most body fluids because they are protected from degradation and external impact (Xiao et al., 2019). Therefore, exosomes have great potential as liquid biopsy specimens for various diseases (Wang et al., 2017; Liu et al., 2019). In particular, cancer cells secrete significantly more exosomes than normal cells (Mousavi et al., 2019; Nabariya et al., 2020). Cancer-derived exosomes likely serve as new circulating biomarkers for the early detection of cancer as they carry cargo reflective of genetic or signaling alterations in the cancer cells of origin (Li et al., 2015; Melo et al., 2015). Therefore, exosomes may be an ideal candidate to act as a biomarker for CRC.

In the present study, we identified several differentially expressed exosomal genes from public databases to understand the underlying molecular changes and biological mechanisms. For the diagnosis of CRC patients, we established a 6–exosomal gene diagnosis model using the least absolute shrinkage and selection operator (LASSO), support vector machine recursive feature elimination (SVM-RFE), and logistic regression analyses. This model was verified using a receiver operating characteristic (ROC) curve in internal and external sets. In addition, the co-expression analysis was used to cluster co-expression modules, and the enrichment analysis was performed on module genes. We also established protein–protein interaction (PPI) networks to investigate hub genes and constructed competing endogenous RNA (ceRNA) networks related to serum exosomal genes in CRC. The purpose of the present study was to obtain further insight into the underlying functions of exosomal genes and to identify any potential diagnostic exosomal genes using the bioinformatics analysis in CRC.

## Materials and Methods

### Data Source and Identification of Differentially Expressed Exosomal Genes

The flowchart of this study is presented in Supplementary Figure S1. We extracted serum exosomal data relative to mRNA, lncRNA, and circRNA from 35 CRC patients and 118 healthy persons from the exoRBase 2.0 database (Li S. et al., 2018) (http://www.exorbase.org/). We also downloaded serum exosomal data from the Gene Expression Omnibus (GEO) database (http://www.ncbi.nlm.nih.gov/geo/). The GSE100063 and GSE100206 datasets contain exosomal data (mRNA) from 12 CRC patients and 32 normal persons. All CRC and healthy exosomal data were available and included in the study. After data normalization and log base 2 transformations, differentially expressed genes between CRC patients and healthy individuals were identified using the limma R package (Ritchie et al., 2015). Differentially expressed genes were defined as those whose expression differences were associated with an adjusted *p* <0.05. This study was approved by the Ethics Committee of the First Affiliated Hospital of Sun Yat-sen University [Approval number (2021)687].

### Identification of the Diagnosis-Related Exosomal Gene Signature Associated With Colorectal Cancer

Genes that were differentially expressed (adjusted *p* <0.05) in the exoRBase 2.0, GSE100063, and GSE100206 datasets were selected. First, 70% of the samples in exoRBase 2.0 were randomly selected as a training set. The LASSO regression analysis was used to obtain diagnostic exosomal genes from the training set. The SVM-RFE analysis was used simultaneously to screen exosomal genes in the training set for CRC diagnosis. Then, we combined the LASSO and SVM-RFE analyses to obtain the common exosomal genes. Next, we selected common exosomal genes that were regulated (up- or downregulated) in the same direction to build a diagnosis-related exosomal gene signature by the multivariate logistic regression analysis. Finally, the ROC curve analysis and the area under the curve (AUC) were used to estimate the diagnostic value of the diagnosis-related exosomal gene signature using the pROC package in R (Robin et al., 2011).

### Validation of the Diagnosis-Related Exosomal Gene Signature

As a validation set, 30% of the samples in exoRBase 2.0, GSE100063, and GSE100206 datasets were selected. To validate whether candidate exosomal genes had an important diagnostic value in patients with CRC, we also measured the ROC curve and the AUC value in the validation datasets. *p* <0.05 is considered statistically significant.

### Cell Culture, Human Plasma Samples, and Isolation of Exosomes

HCoEpiC, HCT116, and SW480 cell lines (purchased from ATCC) were cultured in a DMEM medium (Cellmax) containing 10% fetal calf serum, and 100 U/ml each of penicillin and streptomycin at 37°C with 5% CO_2_. HCoEpiC, HCT116, and SW480 cells were cultured with a full medium at 80% confluency and replaced with a fresh medium without fetal bovine serum. After a 48-h culture, the cell medium was harvested. In addition, thirty-two CRC patient serums and seventeen healthy human serums were collected in the First Affiliated Hospital of Sun Yat-sen University in December 2021. The samples were stored at −80 before exosome extraction. The exosomes were collected from the cell culture supernatant by differential centrifugations. In addition, exosome morphology was identified by transmission electron microscopy (TEM), the nanoparticle tracking analysis (NTA), and the expression of exosome surface markers CD9, TSG101, and HSP70 were evaluated by the Western blotting analysis.

### Validation of Exosomal Gene Expression Levels

Total RNA from exosomes was extracted using the TRIzol reagent (Invitrogen, NYC, United States) and reverse transcribed with the PrimeScript RT kit (Takara, China). Real-time PCR was carried out using the SYBR PreMix Ex Taq II kit (Takara, China). GAPDH was used as the normalized control of mRNA. The relative expression levels of mRNA in exosomes were calculated by the 2^−ΔΔCT^ method. The primer sequences used in this study are listed in Supplementary Table S1.

### The Co-Expression Analysis

The weighted gene co-expression network analysis (WGCNA) was used to identify the co-expression network of differentially expressed exosomal genes in exoRBase 2.0 using the WGCNA package in R (Langfelder and Horvath, 2008). A weighted adjacency was constructed by calculating using the Pearson correlations of all gene pairs. Soft power β = 7 was selected to construct a standard scale-free network. The similarity matrix, which was constructed using the Pearson’s correlation coefficients of all gene pairs, was transformed into a topological overlap matrix (TOM) as well as the corresponding dissimilarity (1-TOM). Then, a hierarchical clustering dendrogram of the 1-TOM matrix was used to classify similar gene expressions into different gene co-expression modules. Afterward, a module-clinical trait association was calculated to identify functional modules in a co-expression network. The brown and gray modules were selected for further analysis.

### The Gene Ontology Term and Kyoto Encyclopedia of Genes and Genomes Functional Enrichment Analyses

To further clarify the potential biological functions of the exosomal genes in the modules, we performed the Gene Ontology (GO) term and Kyoto Encyclopedia of Genes and Genomes (KEGG) pathway analyses using the clusterProfiler package in R (Yu et al., 2012). The GO terms and KEGG pathways with *p* <0.05 were considered significantly enriched.

### Protein–Protein Interaction Network Construction and Hub Gene Identification

In this study, the exosomal genes in the brown and gray modules were analyzed. The PPI network was constructed by the online STRING database (https://string-db.org), and interaction with a combined score >0.7 was considered statistically significant. Cytoscape, an open-source bioinformatics software platform, was used to visualize molecular interaction networks. Hub genes were identified using the Degree algorithm of the cytoHubba plugin in Cytoscape.

### Exosomal ceRNA Network Construction

The TargetScan database (www.targetscan.org) and miRanda (http://cbio.mskcc.org/microrna_data/miRanda-aug2010.tar.gz) statistical models that predict the effects of miRNAs binding to canonical sites of mRNA were used to accomplish miRNA prediction. miRNA/circRNA and miRNA/lncRNA interactions were predicted using ENCORI (http://starbase.sysu.edu.cn/) and miRcode (http://www.mircode.org/), respectively. The ceRNA regulatory network was constructed according to the ceRNA regulatory mechanism and the differentially expressed lncRNAs, circRNAs, and mRNAs in exosomes, and the network were visualized by Cytoscape.

## Results

### Identification of Differential Expression of Exosomal Genes

**FIGURE 1:**
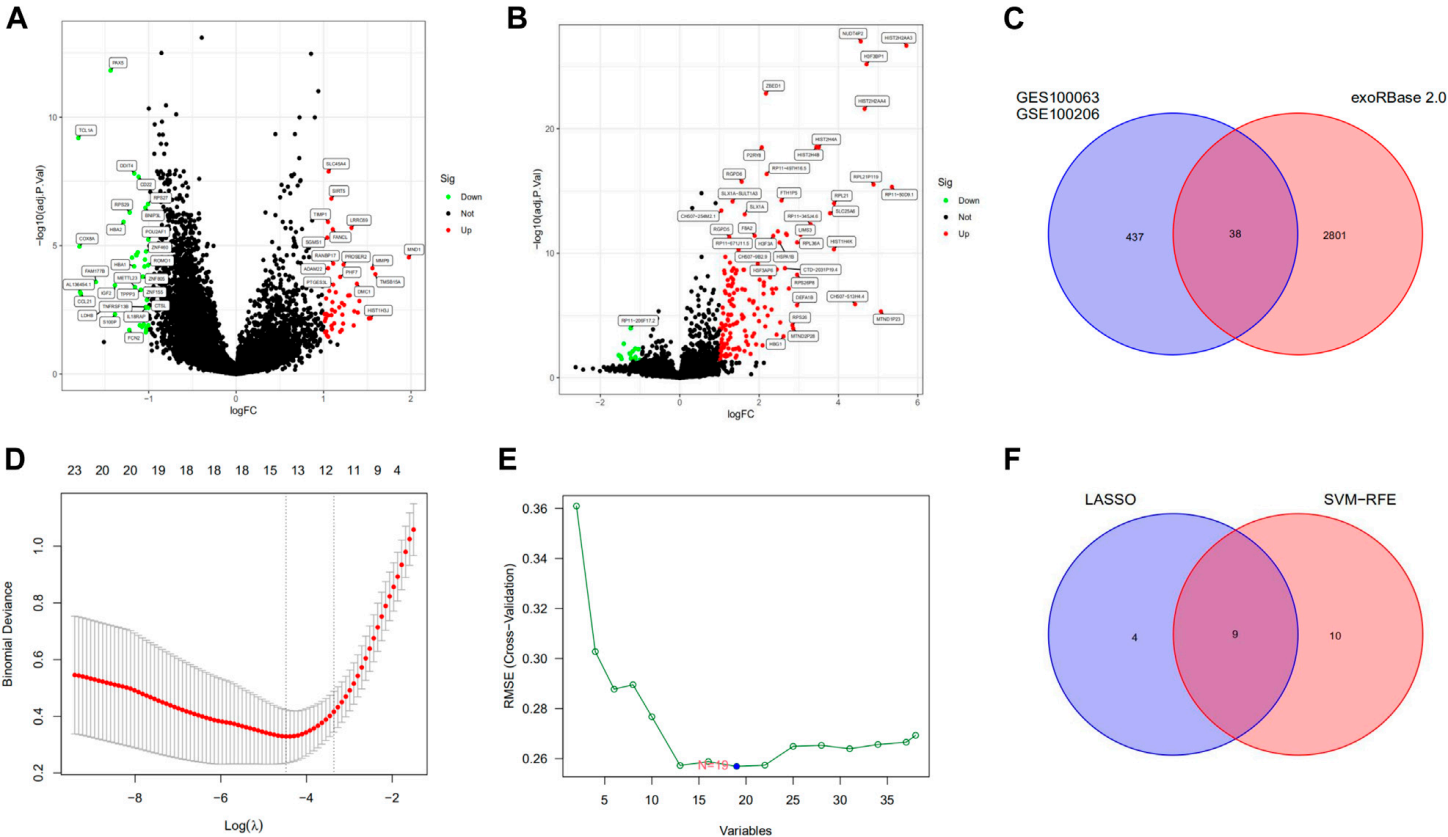
Differentially expressed exosomal genes and identification of diagnostic exosomal genes in CRC. **(A)** Differentially expressed exosomal genes between CRC patients and controls in the exoRBase 2.0 database. **(B)** Exosomal genes differentially expressed between CRC patients and controls in the GSE100063 and GSE100206 datasets. **(C)** The intersection of differentially expressed exosomal genes in the exoRBase 2.0, the GSE100063, and the GSE100206 dataset. **(D)** The LASSO method identified 13 diagnostic exosomal genes. **(E)** The SVM-RFE method identified 19 diagnostic exosomal genes. **(F)** The intersection of diagnostic exosomal genes in the two analyses. CRC, colorectal cancer; LASSO, least absolute shrinkage and selection operator; SVM-RFE, support vector machine recursive feature elimination.

The RNA sequencing exosomal data for CRC and healthy samples were downloaded from the exoRBase 2.0 and GEO databases. A total of 2839 differentially expressed exosomal genes were obtained from the exoRBase 2.0 dataset (Figure 1A) and 475 differentially expressed exosomal genes in the GSE100063 and GSE100206 datasets (Figure 1B). A total of 38 differentially expressed exosomal genes were common in the two databases (Figure 1C).

### Development and Verification of the Diagnostic Model

**FIGURE 2:**
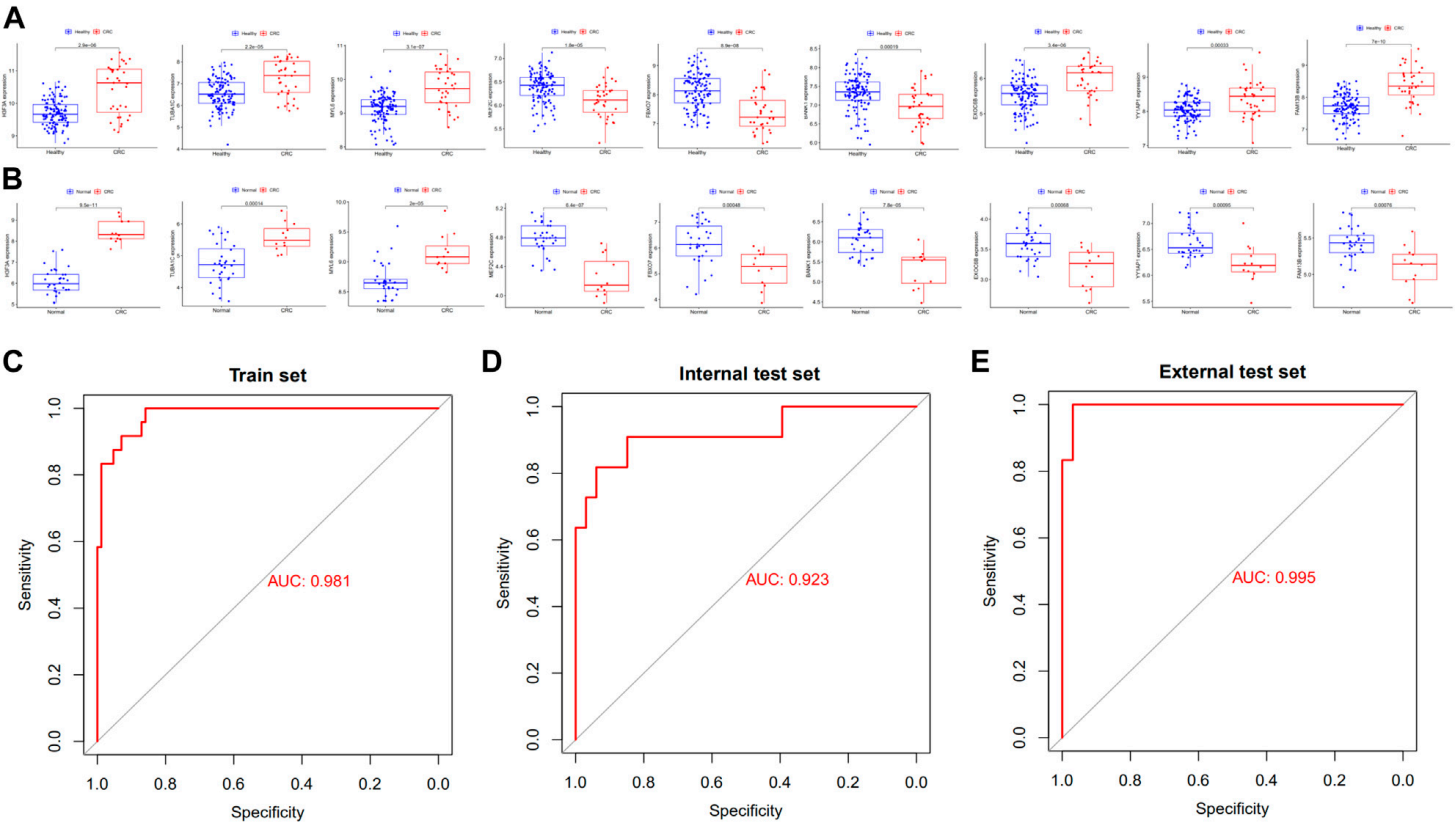
Potential exosomal genes for the diagnosis of CRC. The relative expression level of diagnostic exosomal genes **(A)** between CRC serum and healthy samples in the training set and **(B)** in the external validation dataset. ROC curves of the exosomal gene signature in the **(C)** training set and **(D)** in the internal validation set of the exoRBase 2.0 database. **(E)** ROC curves of the exosomal gene signature in the external validation set of the GSE100063 and GSE100206 database. CRC, colorectal cancer; ROC, receiver operating characteristic.

We randomly split all the samples in the exoRBase 2.0 dataset into a training set (70%) and a validation set (30%). We combined the LASSO and SVM-RFE methods to obtain 9 common exosomal genes (Figures 1D–F). Next, in the training and validation set, we selected common exosomal genes that were in the same regulated direction to build a diagnosis-related exosomal gene signature using the multivariate logistic regression analysis (Figures 2A,B). Therefore, we obtained 6 exosomal genes including *H3F3A*, *MYL6*, *FBXO7*, *TUBA1C*, *MEF2C*, and *BANK1*. Furthermore, these 6 exosomal genes were identified based on the model according to the following formula: index = *H3F3A* × (3.67971578945741) + MYL6 × (2.01995321634272) + FBXO7 × (−0.86536340517915) + TUBA1C × (0.184108184598825) + MEF2C × (−3.86524121779742) + BANK1 × (−5.35388054514927). The AUC for the gene signature was 0.981 in the training set (Figure 2C) and 0.923 in the internal test set (Figure 2D). Importantly, we also used external datasets to validate the gene signature: it showed good diagnostic ability, with an AUC of 0.995 (Figure 2E).

### Validation of Exosomal Gene Expression Levels

**FIGURE 3:**
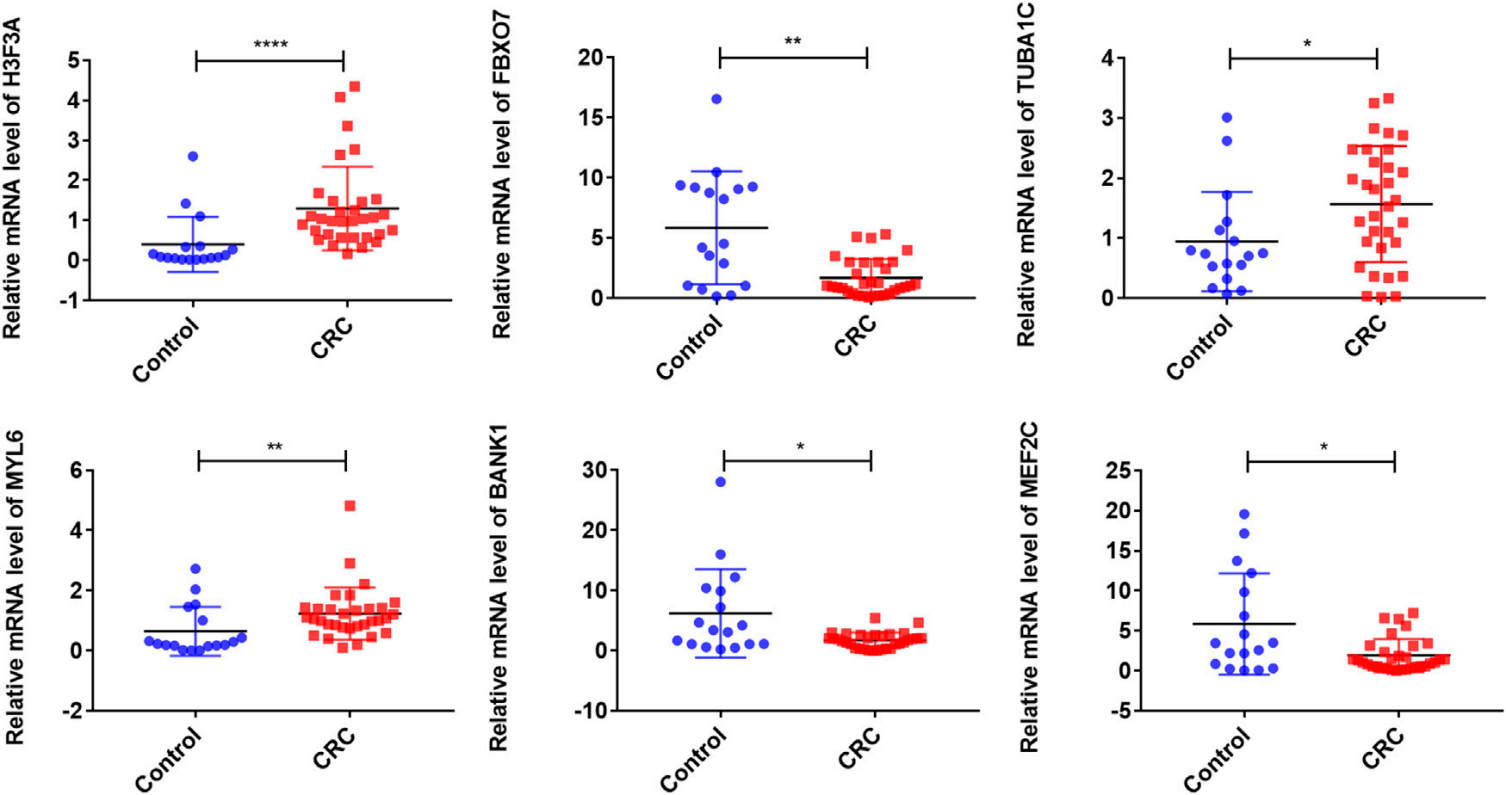
Validation of exosomal gene expression levels in CRC patient serums and controls. ∗∗∗∗*p* < 0.0001; ∗∗*p* < 0.01; ∗*p* < 0.05; CRC, colorectal cancer.

To further validate the expression levels of these model exosomal genes, we extracted exosomes from HCoEpiC, HCT116, SW480 cells, and human plasmas. The purification of the exosomes was validated by TEM, NTA, and Western blotting analysis. TEM detected double-layer spherical vesicles ranging from 30 to 160 nm in size, which confirmed the presence of exosomes (Supplementary Figure S2A). NTA characterized the size and concentration of exosomes (Supplementary Figure S2B). Western blotting analysis estimated the quantity and purity of exosomes by detecting exosomal marker proteins (CD9, TSG101, and HSP70) (Supplementary Figure S2C). Next, we validated the expression of signature exosomal genes in the exosomes obtained by RT-qPCR. The results showed that *H3F3A*, *MYL6*, and *TUBA1C* were significantly upregulated in CRC cells, and *MEF2C* and *FBXO7* were significantly downregulated in CRC cells. However, no significant differences were observed in *BANK1* expression between HCT116 and SW480 cells and HCoEpic cells (Supplementary Figure S2D). Furthermore, we validated the expression levels of the model exosomal genes in the serums from the CRC patients and healthy humans. The results showed that the mRNA levels of three genes (*H3F3A*, *TUBA1C,* and *MYL6*) were significantly elevated in the CRC exosomes, whereas *BANK1*, *MEF2C,* and *FBXO7* were downregulated in the CRC exosomes when compared with those in the healthy human exosomes (Figure 3). These results were consistent with the results of the exoRBase 2.0 and GEO databases.

### The Weighted Gene Co-Expression Network Analysis and Key Module Identification

**FIGURE 4:**
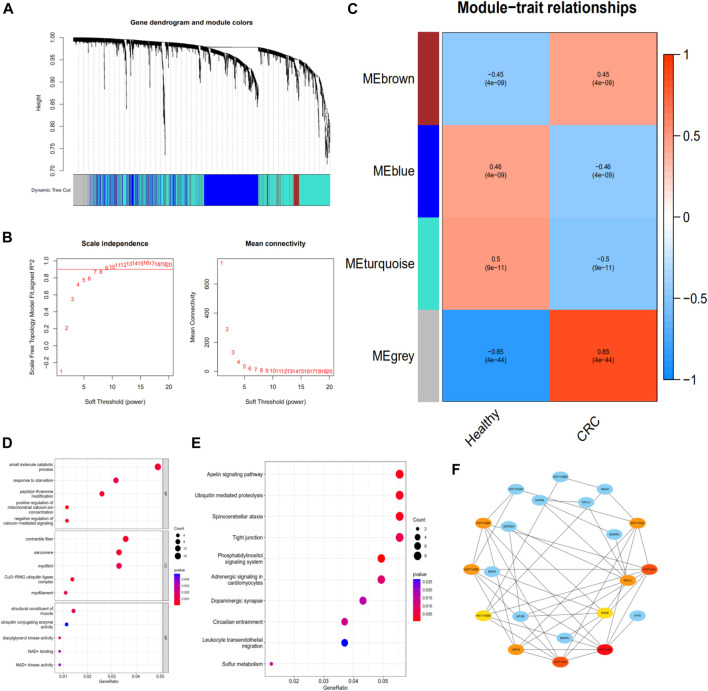
Identifying the functions of CRC-associated exosomal genes. **(A)** Clustering dendrogram. **(B)** Determination of soft-thresholding power in the weighted gene co-expression network analysis. **(C)** Module–trait associations evaluated by correlations between CRC and clinical traits. **(D)** The GO enrichment analysis of the exosomal genes in the brown and gray modules. **(E)** The KEGG pathway of the exosomal genes in the brown and gray modules. **(F)** The PPI network of genes in the brown and gray modules and hub genes screening. CRC, colorectal cancer; PPI, protein–protein interaction

To identify exosomal genes associated with CRC, we analyzed differentially expressed exosomal genes between CRC and healthy samples in the exoRBase 2.0 database using WGCNA. The included samples were clustered with the hierarchical average linkage clustering method (Figure 4A). The optimal soft power threshold for WGCNA was set to 7 to preserve the scale-free topology and effective connectivity (Figure 4B). Four co-expression modules of differentially expressed exosomal genes were established (Figure 4C). The brown and gray modules were found to have a positive correlation with CRC.

### Enrichment of Module Genes

To explore the potential function of exosomal genes in the brown and gray modules, the enrichment analysis of GO and KEGG was performed. The GO analysis of these differentially expressed exosomal genes revealed that “small molecule catabolic process,” “contractile fiber,” and “structural constituent of muscle” were the most frequent biological terms for biological process, cellular components, and molecular functions, respectively (Figure 4D). The KEGG analysis revealed that these exosomal genes were mainly enriched in the “apelin signaling pathway,” “ubiquitin mediated proteolysis,” “spinocerebellar ataxia,” and “tight junction” (Figure 4E).

### Protein–Protein Interaction Network Construction and Hub Genes Screening

The PPI network of the exosomal genes in the brown and gray modules was constructed through the STRING database and visualized with Cytoscape. The PPI network and hub genes identified from the network were obtained through the degree algorithm of the CytoHubba plugin. According to degree scores, the top-scoring genes, including *HIST1H3E*, *HIST1H3J*, *HIST1H3A*, *HIST1H2BC*, *SEH1L*, *H3F3A*, *HIST1H2BJ*, *HIST1H2BF*, *HIST1H2BB*, and *RHEB*, were considered the hub genes (Figure 4F).

### Construction of the Exosomal ceRNA Network

**FIGURE 5:**
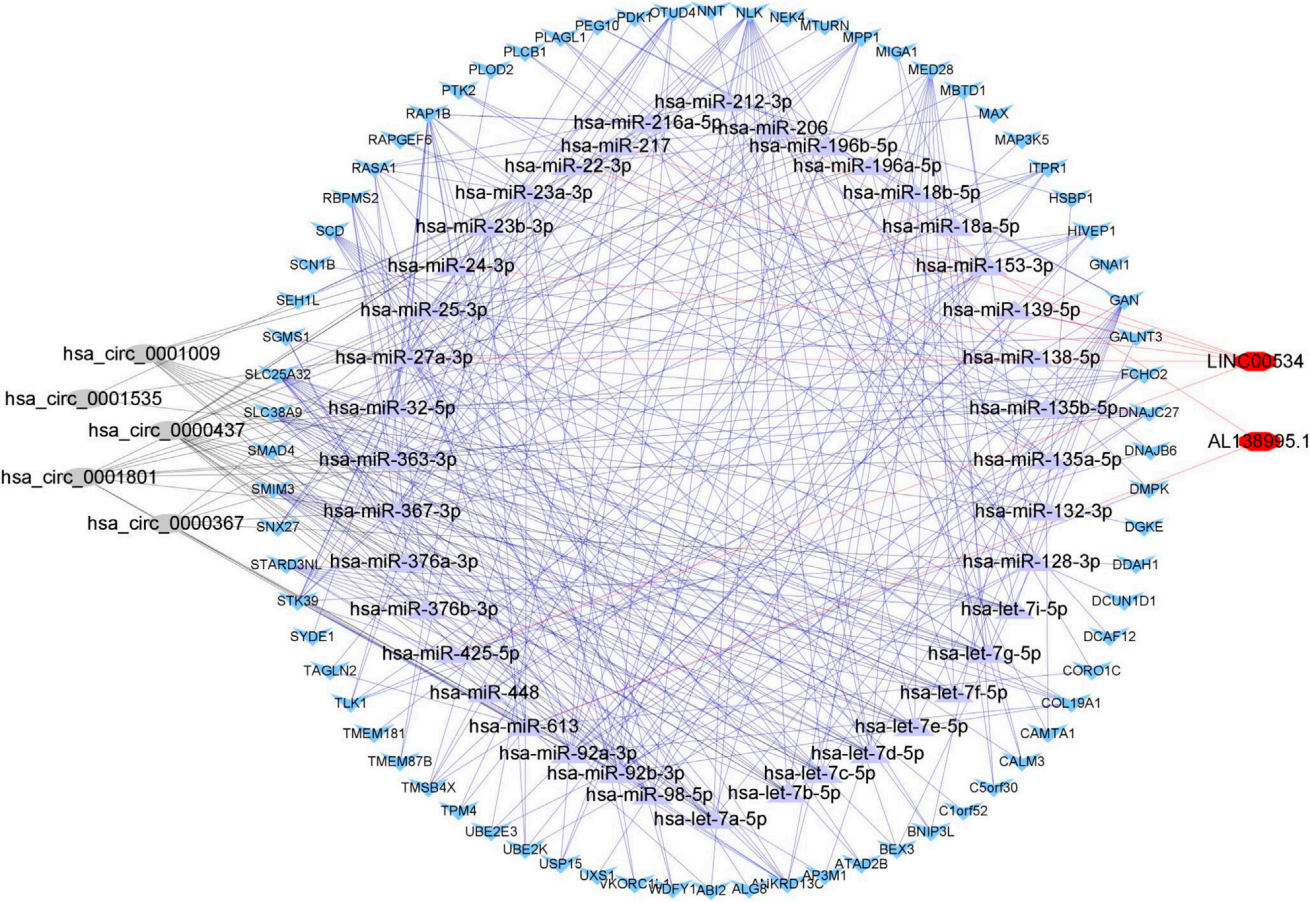
A competing endogenous RNA network associated with exosomal genes.

After the differentially expressed exosomal genes were identified in the brown and gray co-expression modules, these exosomal genes were used to construct ceRNA networks. Exosomal lncRNA and circRNA that were differentially expressed (adjusted *p* <0.05) in exoRBase 2.0 were selected. Based on mRNA, lncRNA, circRNA, and the predicted corresponding miRNA, we constructed a ceRNA network. The ceRNA network consisted of 5 circRNA nodes, 2 lncRNA nodes, 40 miRNA nodes, and 72 mRNA nodes (Figure 5).

## Discussion

CRC is a highly malignant cancer with a poor prognosis. CRC patients are at substantial risk of recurrence and metastasis. Therefore, early diagnosis is very important to improve the clinical prognosis of CRC patients. Liquid biopsy, a recent and hot topic in cancer detection, has been considered for the early diagnosis of cancer (Chang et al., 2019). The functional states of cancer cells could be assessed by testing the exosome they secreted, which provides the basis for exosome-mediated noninvasive cancer liquid biopsy (Yang et al., 2020). Currently, various studies show that the increased or decreased expression of exosomes plays an important role in different kinds of cancer, including CRC (Hon et al., 2017; Galamb et al., 2019; Xiao et al., 2020). Thus, exosomes are crucial potential candidates for the early detection of CRC.

To date, exosomal gene–based diagnostic models have not been described for CRC, although considerable efforts have been made to develop prognostic signatures based on differentially expressed genes (Chen et al., 2019; Dai et al., 2020; Wang et al., 2020). The present study analyzed differences in exosomal gene expression between CRC patients and healthy humans. Importantly, we identified diagnostic exosomal genes based on a comprehensive analysis that could serve as valuable biomarkers in the clinical setting.

Potential exosomal gene modules related to CRC were identified with the WGCNA analysis. The brown and gray modules were found to have a positive correlation with CRC. To better understand the potential function of exosomal genes among the brown and gray modules, the GO and KEGG enrichment analysis and the PPI network were conducted. The results of the functional and pathway enrichment analyses showed that the exosomal genes in the modules were mainly enriched in the structural components of the muscle and apelin signaling pathway. The PPI network of exosomal genes in the brown and gray modules was constructed and 10 hub genes were selected using the CytoHubba plugin in Cytoscape. Among these genes, H3F3A, as a diagnostic biomarker in the present study, was also identified as a hub gene, and maybe as a promoter of CRC progression and metastasis. The ceRNA network played a critical role in the initiation and progression of CRC (Ke et al., 2019; Ma et al., 2020). In the present study, we further constructed ceRNA networks based on those key exosomal genes. This approach provided a novel view of the RNA–RNA crosstalk in the exosome and indicated the potential diagnostic and therapeutic functions of exosomal ceRNA networks in CRC.

Our exosomal gene–based model highlighted 6 exosomal genes, that is, *H3F3A*, *MYL6*, *FBXO7*, *TUBA1C*, *MEF2C*, and *BANK1*. These genes and their biological functions have been studied in some tumors. *H3F3A* is one of two genes encoding histone H3.3, a noncanonical histone variant, and has been established as a major driver gene of malignant gliomas (Schwartzentruber et al., 2012; Sturm et al., 2012; Wu et al., 2012). *MYL6*, a gene that encodes hexameric ATPase cellular motor protein, is upregulated in circulating tumor cells of many cancers (Yadavalli et al., 2017). *FBXO7* may have a proto-oncogenic role in epithelial tumors (Laman et al., 2005). *TUBA1C*, as a type of tubulin, is associated with tumor cell death and cell proliferation, and *TUBA1C* overexpression is predicted to have a poor prognosis (Li C.-W. et al., 2018; Li et al., 2020). *MEF2C* was traditionally considered a development-associated factor and can inhibit tumor growth *in vitro* and *in vivo* (Bai et al., 2015). *BANK1*, which encodes a protein adaptor that is predominantly expressed in B cells, is a putative tumor suppressor gene in B-cell lymphomagenesis (Yan et al., 2014). Our results showed that diagnostic exosomal genes may participate in the development of human CRC; however, the underlying molecular mechanism of these genes in the prognosis of CRC requires further investigation. Our experimental results showed that the differential expression levels of diagnostic exosomal genes in cell lines were approximately in agreement with the serum exosomal data in these public databases.

To the best of our knowledge, this is the first reported exosomal gene–based model for CRC. However, the potential limitations of this study should also be considered when interpreting the findings. First, we could not explore the association between these exosomal genes and the prognosis of CRC patients due to the lack of therapeutic and prognostic information. Second, because the data we analyzed were obtained from public databases, further experimental studies are necessary to validate our findings.

To conclude, we investigated the potential functions and diagnostic values of exosomal genes in CRC through a comprehensive bioinformatics analysis. A diagnostic exosomal gene model was constructed. This model can assess the value of exosomal genes to diagnose CRC using a noninvasive method and might be useful for the development of individualized treatment for CRC patients, but the feasibility of its use in the population needs to be further validated.

## Data Availability

The original contributions presented in the study are included in the article/Supplementary Material; further inquiries can be directed to the corresponding author.

## Supplementary Material

Supplementary Figure S1 Overall workflow of the present study.

Supplementary Figure S2 Identification of exosomes and validation of signature exosomal gene expression levels. **(A)** Morphologies of exosomes in HCoEpic, HCT116, and SW480 cell lines were observed by transmission electron microscopy. **(B)** The size and concentration of exosomes by the nanoparticle tracking analysis. **(C)** Exosomes were analyzed by Western blotting using antibodies against exosomal markers. **(D)** The relative expression fold of diagnostic exosomal genes was determined. *****P*<0.0001; ****P*<0.001; ***P*<0.01.
