# Supplementary material for: A Diagnostic Model Using Exosomal Genes for Colorectal Cancer
Source: Front Genet. 2022 Jul 15;13:863747. doi: 10.3389/fgene.2022.863747 (PMC9334773; doi:10.3389/fgene.2022.863747)
Supplement: Supplementary file 1 [file Table1.docx]

**Supplementary Table 1 |** The primers of genes.

| **Genes** | **Forward primers** | **Reverse primers** |
| --- | --- | --- |
| MYL6 | 5’-GAAGACCAGACCGCAGAGTTC-3’ | 5’-TCCAGCACCTTCACATTCATC-3’ |
| FBXO7 | 5’-GATTCAGAGCATTCTTCACTCCA-3’ | 5’-GCCCTAACATACTGTCGTCATTC-3’ |
| TUBA1C | 5’-TGTTTGTAGACTTGGAACCCAC-3’ | 5’-GCCAATGGTGTAGTGCCCT-3’ |
| MEF2C | 5’-CTGGTGTAACACATCGACCTC-3’ | 5’-GATTGCCATACCCGTTCCCT -3’ |
| BANK1 | 5’-ACACAGCCCACTAGAGGTTG-3’ | 5’-GGTGAAGTGAGGTCTTTCCAG-3’ |
| H3F3A | 5’-TGTGGCGCTCCGTGAAATTAG-3’ | 5’-CTGCAAAGCACCGATAGCTG-3’ |
| GAPDH | 5’-CAAGGTCATCCATGACAACTTTG -3’ | 5’-GGCCATCCACAGTCTTCTGG -3’ |
