# Supplementary figures and images for: A Diagnostic Model Using Exosomal Genes for Colorectal Cancer
Source: Front Genet. 2022 Jul 15;13:863747. doi: 10.3389/fgene.2022.863747 (PMC9334773; doi:10.3389/fgene.2022.863747)

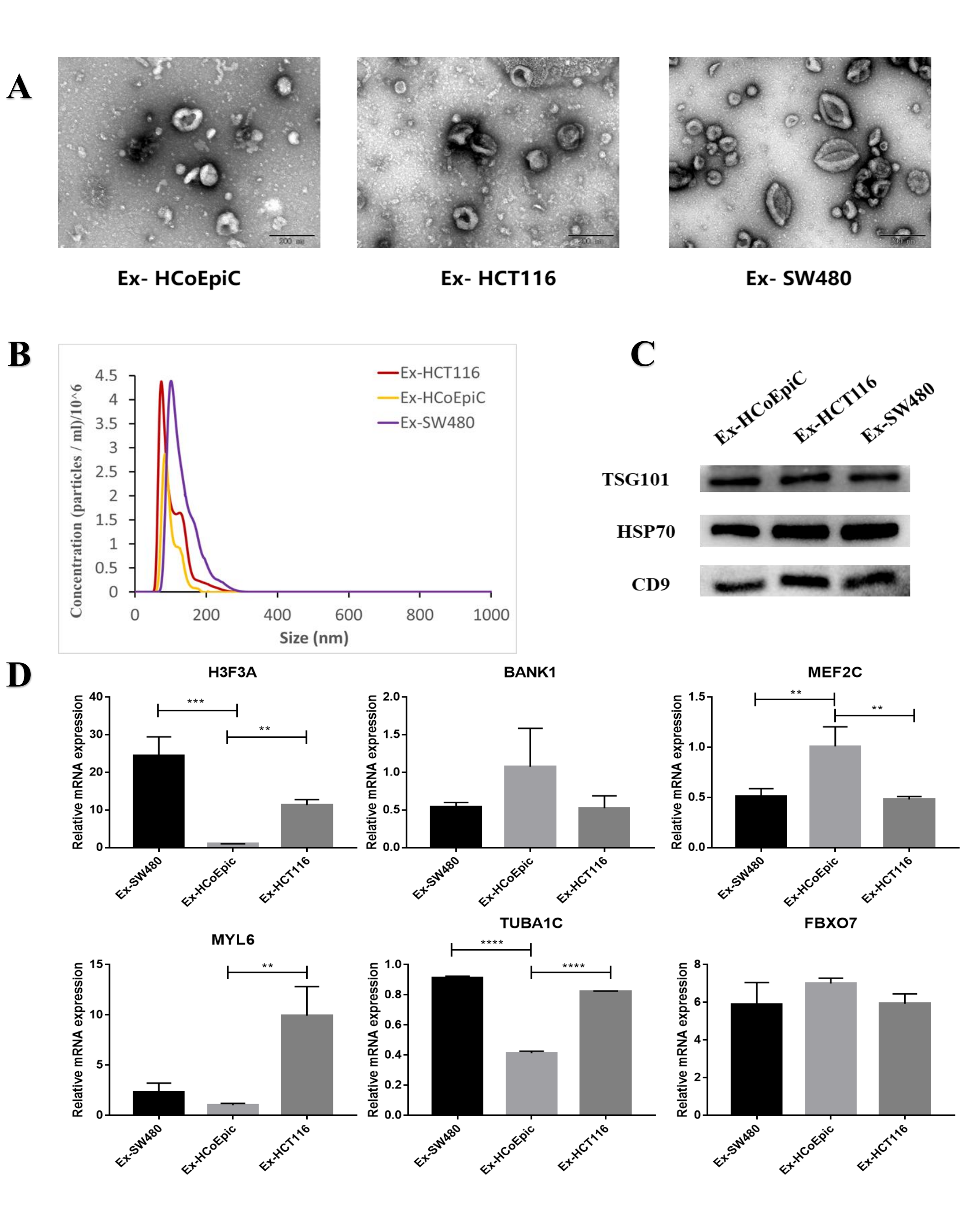

Supplement: Supplementary file 2 [file Image2.tif]

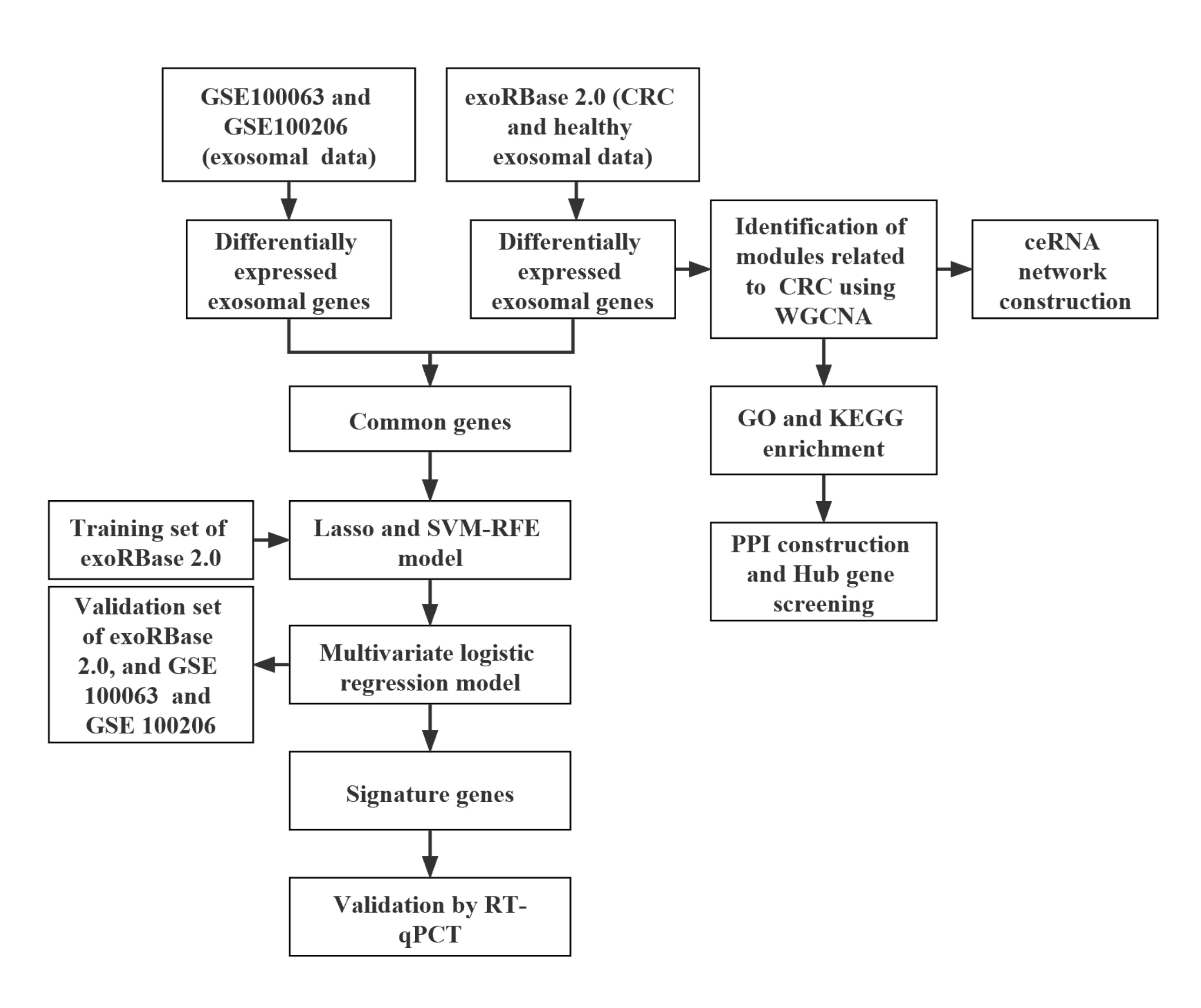

Supplement: Supplementary file 3 [file Image1.tif]
